# The Immunopathology of Giant Cell Arteritis Across Disease Spectra

**DOI:** 10.3389/fimmu.2021.623716

**Published:** 2021-02-25

**Authors:** Michelle L. Robinette, Deepak A. Rao, Paul A. Monach

**Affiliations:** ^1^Division of Rheumatology, Inflammation, and Immunity, Brigham and Women's Hospital and Harvard Medical School, Boston, MA, United States; ^2^Rheumatology Section, VA Boston Healthcare System, Boston, MA, United States

**Keywords:** vasculitis, CIA, LVV, Takayasu, PMR, temporal arteritis, GCA, giant cell arteritis

## Abstract

Giant cell arteritis (GCA) is a granulomatous systemic vasculitis of large- and medium-sized arteries that affects the elderly. In recent years, advances in diagnostic imaging have revealed a greater degree of large vessel involvement than previously recognized, distinguishing classical cranial- from large vessel (LV)- GCA. GCA often co-occurs with the poorly understood inflammatory arthritis/bursitis condition polymyalgia rheumatica (PMR) and has overlapping features with other non-infectious granulomatous vasculitides that affect the aorta, namely Takayasu Arteritis (TAK) and the more recently described clinically isolated aortitis (CIA). Here, we review the literature focused on the immunopathology of GCA on the background of the three settings in which comparisons are informative: LV and cranial variants of GCA; PMR and GCA; the three granulomatous vasculitides (GCA, TAK, and CIA). We discuss overlapping and unique features between these conditions across clinical presentation, epidemiology, imaging, and conventional histology. We propose a model of GCA where abnormally activated circulating cells, especially monocytes and CD4^+^ T cells, enter arteries after an unknown stimulus and cooperate to destroy it and review the evidence for how this mechanistically occurs in active disease and improves with treatment.

## Introduction

Giant cell arteritis (GCA) is a granulomatous systemic vasculitis of people age 50 or older that affects large- and medium-size arteries (1, 2). Vascular inflammation has two major patterns, which overlap in a clinical spectrum. The first and classic pattern, originally described by Horton in 1932, involves inflammation of the extracranial branches of the carotid artery with predilection for the temporal artery and is called cranial GCA. The second pattern involves the aorta and its proximal branches, particularly the axillary, subclavian, and proximal brachial branches, and is called large-vessel GCA (LV-GCA) (3). While autopsy studies in the 1970s demonstrated LV involvement in patients with cranial-GCA (4, 5), advances in imaging in the past decade have reemphasized the frequent co-occurrence of subclinical LV with cranial disease and have identified the less common entities of isolated cranial- and LV-GCA (6). Along with Takayasu arteritis (TAK), a systemic vasculitis that occurs mostly in women under age 50, and clinically isolated aortitis (CIA), a vasculitis restricted to the aorta, GCA is one of three non-infectious granulomatous vasculitides with prominent aortic involvement (1).

GCA is medical emergency due to its ability to cause irreversible vision loss and requires prompt diagnosis and initiation of treatment. Individual patient presentations vary depending on the complement of cranial or large vessels that are involved, yet patients often share common systemic features. These include laboratory evidence of systemic inflammation, constitutional symptoms, and polymyalgia rheumatica (PMR), a condition characterized by pain and stiffness in the neck, shoulders, and pelvic girdle that often co-occurs with GCA (7, 8). Mechanistic understanding of both GCA and PMR has been limited by the lack of consensus diagnostic criteria. However, GCA is better characterized than PMR due to the historical *de facto* diagnostic gold standard being temporal artery biopsy (TAB), which has created a more homogenous clinical group and also provided a vital source of tissue for research purposes. Immunosuppression with glucocorticoids (GC) is the cornerstone of treatment for both GCA and PMR. As most patients have disease flares with GC tapering and require prolonged treatment, steroid sparing agents have been sought, with methotrexate identified as providing benefit in PMR and likely some in GCA, and targeted blockade of IL-6R with tocilizumab (TCZ) providing benefit in GCA. Multiple other drugs are being studied in clinical trials in GCA (9–12).

Here, we review the current understanding of the immunopathology of GCA on the background of the three settings in which comparisons are informative: LV and cranial variants of GCA; PMR and GCA; and the three granulomatous vasculitides (GCA, TAK, and CIA). We also discuss clinical presentation and epidemiology of disease, and the growing role of advanced imaging for clinical and research use. We identify areas of uncertainty and discuss possible mechanisms of disease pathogenesis.

## Clinical Presentation

Systemic inflammation is a cardinal feature of GCA, as well as PMR and TAK. Clinically, many patients experience non-specific constitutional symptoms including fatigue, anorexia, weight loss, fever, and night sweats. Laboratory evidence of inflammation includes anemia, thrombocytosis, and elevations in the inflammatory markers erythrocyte sedimentation rate (ESR) and/or C-reactive protein (CRP). Patients with CIA lack systemic features, according to the most commonly used definition of CIA (7, 8, 13, 14).

Cranial symptoms of GCA are the classic presentation of disease and account for the majority of the 1990 ACR classification criteria (7). Inflammation of medium-size arteries causes pain and tenderness in the artery wall itself and leads to vascular stenosis and ultimately occlusion, causing symptomatic ischemia. Ischemic symptoms include headache, jaw claudication, and acute onset visual disturbances (7), and are inversely correlated with the degree of systemic inflammation (15, 16). More rarely, scalp or tongue necrosis, sensorineural hearing loss, and even vertebrobasilar stroke can occur. The most commonly feared complication is irreversible vision loss, which occurred in 15–35% of patients prior to widespread recognition of GCA and emergent use of GC (2, 17, 18).

LV-GCA often presents with non-specific systemic symptoms, leading to delayed diagnosis (19, 20). Features suggestive of LV-GCA in patients with PMR include the need for unusually high doses of GC, bilateral diffuse lower extremity pain, pelvic girdle pain, and inflammatory low back pain (20). LV-GCA can also cause ischemic symptoms corresponding to supra-aortic vessel stenosis with resultant limb claudication or dizziness. Physical signs can include vascular bruits, loss of carotid or radial pulses, and/or discordant blood pressures (19, 21). These overlap with the symptoms and classification criteria for TAK (13). Rather than causing ischemia in downstream organs, inflammation of the aorta under the stress of high-pressure gradients generated by the heart leads to dilatation in 32% of patients with GCA, aneurysm formation in 2–10% patients, and ultimately may progress to dissection (22–24). Thus, LV-GCA is typically identified on imaging or in surgical specimens from repairs of aneurysms or dissections. In the case of surgical tissue, GCA must further be differentiated from CIA by evidence of systemic features or evidence of disease in arteries other than the aorta.

## Epidemiology

GCA is the most common form of vasculitis in patients over age 50 with most being much older. PMR is 3–10 times more common than GCA and is the second most frequent rheumatic disease of elderly after rheumatoid arthritis (2). Forty–sixty percent of patients with GCA have symptoms of PMR while 16–21% PMR have GCA (25, 26). Age >50 is a defining feature of both GCA and PMR, and both peak around age 75, with the exception that patients with LV-GCA are typically younger between 50 and 65 (2, 3, 24, 27, 28). Other granulomatous vasculitides affecting the aorta also occur earlier; CIA has a mean diagnosis of age 65 while TAK peaks between 15 and 29 (14, 29). All conditions are more common in women, with increasing frequency from CIA and PMR (2:1), to cranial-GCA (almost 3:1), to LV-GCA (3:1), and finally to TAK (9:1) (3, 14, 26–30).

The incidence of cranial-GCA and PMR is most frequent in patients of Northern European ancestry. Overlapping incidence of GCA between Northern Europe at 14.6–43.5/ 100,000 and the ancestrally similar Olmstead County, Minnesota at 19.8/ 100,000 suggest a genetic predisposition (26, 27). In other populations, GCA occurs between 1.1 and 11.1/100,000 (26, 31–33), though there are no studies from Africa, South America, or the majority of continental Asia and the Middle East. It was previously thought that GCA was uncommon in African Americans (31). However, this has not consistently been shown in the literature, likely reflecting the ancestral heterogeneity within racial groups within the United States and perhaps under recognition of GCA in African Americans due to the misconception they are not affected (34–38). TAK is less common than GCA, with highest incidence in Asia, South America, and Turkey at 1–2/1,000,000 (39). The true incidence and demographics of LV-GCA and CIA are unknown but appear to be intermediate between cranial-GCA and TAK, at least in the United States (40).

While the increased frequency of GCA in patients of Northern European ancestry suggests a genetic predisposition, genetic studies have generated limited insight into pathophysiology of disease. An early reported and consistently reproduced finding is the association with MHC class II HLA DRB04, specifically the ^*^0401 and ^*^0404 alleles, with cranial- and LV-GCA as well as PMR (3, 41–44). Indeed, large immune-focused genotyping arrays performed on patients with TAB-confirmed cranial-GCA and TAK identified the HLA locus as the only locus to achieve genome-wide significance for association with GCA, and one of two loci with genome-wide significance in TAK (45, 46). In GCA, the majority of this association was due to HLA-DRB1 and HLA-DQA1, with a minor contribution from MHC class I HLA-B. The opposite pattern was found for TAK (47). Strong class II associations suggest a key role for antigen presentation by MHC class II to helper CD4^+^ T cells in GCA, and multiple studies have suggested changes to the MHC class II peptide-binding groove, however, the specific antigens recognized by CD4^+^ T cells in GCA remain unclear (41, 46). Likewise, TAK has more cytotoxic CD8^+^ T cell infiltration than GCA that may explain its association with class I (48). When data from GCA and TAK studies were combined in a meta-analysis, the only non-HLA SNP that reached significance was in IL12B, encoding the p40 portion of the IL-12 (p35p40)/IL-23 (p19p40) heterodimeric proteins that is shared by both cytokines (47). Yet, clinically targeting p40 with ustekinumab in two open-label trials has shown mixed results in GCA (49, 50). Collectively, epidemiologic data emphasizes the importance of old age, female sex, and genetics with GCA though how these factors contribute to disease pathogenesis remains largely unclear.

## Imaging

In 2018 the European League Against Rheumatism (EULAR) issued guidelines for use of imaging in LVV for the first time, recommending early imaging as the diagnostic test of choice to replace TAB in all cases of clinically suspected GCA (51). Currently, there are four major imaging modalities used in clinical practice (Table 1): ultrasound, MRI, CT, and [^18^F]-fluorodeoxyglucose (FDG) positron emission tomography (PET) (6, 51). PET is combined with another technique, most often CT. All four modalities assess vascular wall thickness and a marker of inflammation that differs between techniques (Table 1). Ultrasound assessment is limited to superficial arteries and patients with GCA have non-compressible hypoechoic wall thickening called the “halo sign” (51). Compared to ultrasound, MRI angiography and CT angiography have increased vascular resolution, facilitating assessment of luminal irregularities such as vascular stenosis, aneurysm, and occlusion. Special MRI contrast sequences can also assess cranial vasculature (55, 56).

**Table 1:** Characteristics of imaging modalities clinically used to support a GCA diagnosis.

	**Ultrasound**	**MRI**	**CT**	**FDG-PET**
**Vasculature examined**	Superficial cranial, carotid, and axillary arteries	Cranial arteries, all large arteries	All large arteries	All large arteries; emerging use in cranial arteries
**Marker of inflammation**	“Halo sign” —vascular edema	Contrast enhancement	Contrast enhancement	FDG uptake—glucose metabolism
**Advantages**	Low cost, non-radiating	Vascular resolution, non-radiating	Vascular resolution, second lowest cost	Exam sequence not limited to vasculature and may detect mimics such as cancer, emerging use in flare
**Disadvantages**	Operator dependent, limited to superficial arteries	Reduced accessibility, high cost, highest number of patient contraindications	Reduced accessibility, radiation	Lowest accessibility, highest cost, radiation when combined with CT
**EULAR GCA recommendation**	Cranial-GCA, LV-GCA	Cranial-GCA, LV-GCA	LV-GCA	LV-GCA
**Sensitivity^a^**	Pooled: 77%^b^ (95% CI: 62–87%)	Pooled: 73%^b^ (95% CI: 57–85%)	73%^c^	67–71%^d^
**Specificity^a^**	Pooled: 96%^b^ (95% CI: 85–99%)	Pooled: 88%^b^ (95% CI: 81–92%)	85%^c^	91–100%^d^

a *Compared to clinical diagnosis of GCA. Caveats include that clinical diagnosis by ACR criteria favors cranial-GCA and there are fewer studies prospectively assessing sensitivity and specificity of CTA and PET*.

b *Data from a recent meta-analysis (52)*.

c *(53)*.

d *(53, 54)*.

PET is a very sensitive technique that detects inflammation through the surrogate marker of increased glucose metabolism *via* FDG uptake and has been particularly important to define GCA and PMR. In 1999, a small prospective PET study first demonstrated LV enhancement in patients with GCA and, surprisingly, equally in those with PMR (57). Most subsequent studies are retrospective raising the possibility of selection bias. However, additional small prospective PET studies have demonstrated LV FDG uptake in 66.7–83% of patients with cranial GCA (54, 58) and 14–31% of patients with PMR (59, 60). Corresponding to limb girdle symptoms, patients with PMR show additional FDG uptake in periarticular regions to the hip and shoulder, ischial tuberosities, sternoclavicular joints, and trochanteric and interspinous bursa (61). Pathologic correlates to large vessel imaging studies are not intentionally obtained. Supporting the concept that imaging findings do reflect active vascular inflammation, some studies have reported that inflammatory markers correspond to the degree of LV FDG uptake (54, 62, 63), which is also reduced with treatment (58, 64, 65). However, low grade enhancement may persist with normal inflammatory markers (66). Multiple prospective serial studies have now shown this does not appear to predict clinical relapse and may rather represent vascular remodeling (58, 65, 67). Preliminary data suggests there may be an imaging cut-off that can distinguish ongoing inflammation from vascular remodeling, as well as from LVV mimics such as atherosclerosis, and is an ongoing area of research (68, 69).

**Figure 1:**
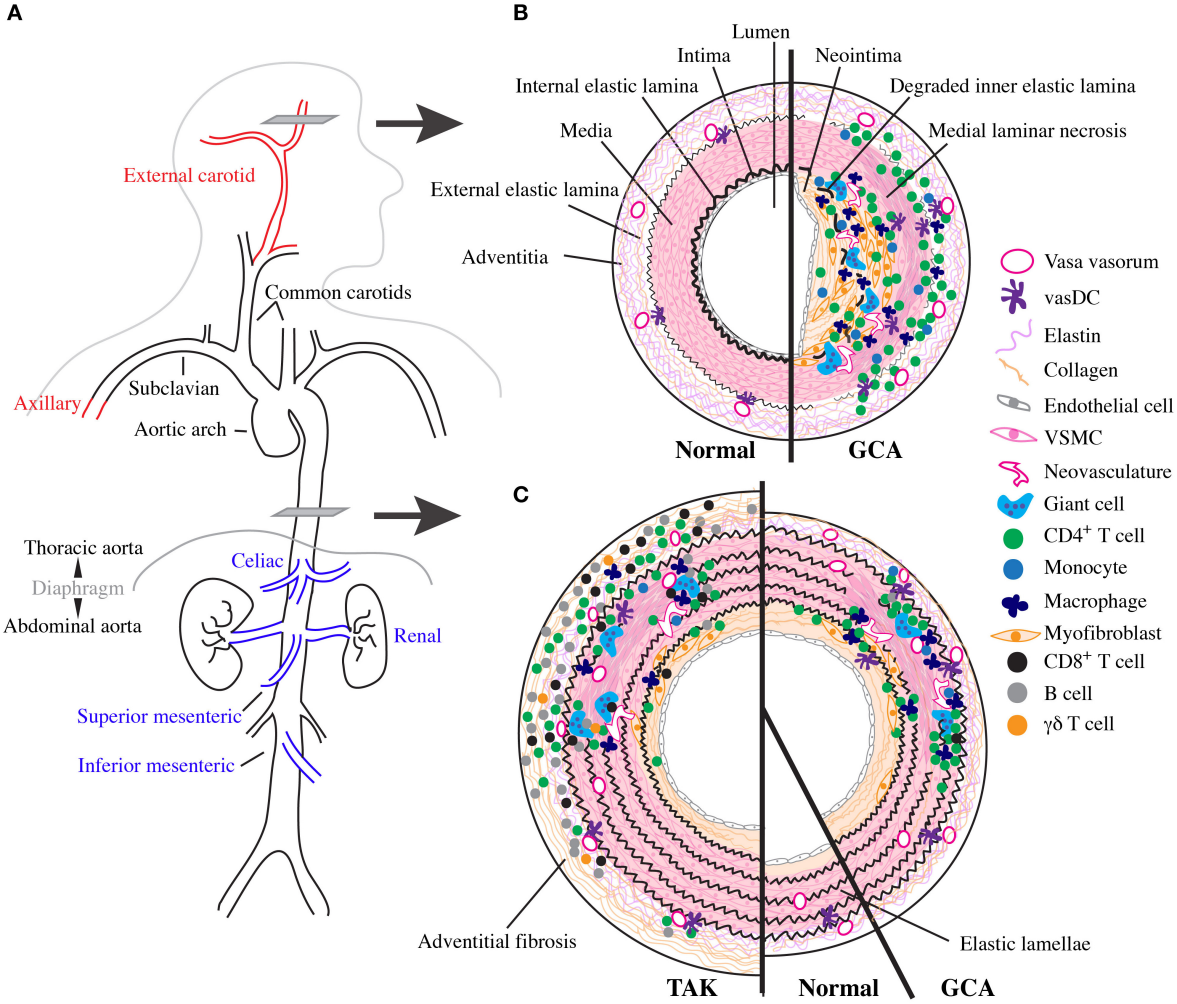
Vascular topology and schematic cross sections of GCA, TAK, and normal arteries. **(A)** Depiction of the normal human large vessel vasculature. Vascular involvement with greatest specificity for GCA shown in red and with greatest specificity for TAK in blue. **(B)** Cartoon representing a cross section though the temporal artery as indicated in **(A)** with normal vessel on the left and the inflammatory infiltrate and vascular remodeling found in cranial-GCA on the right. **(C)** Cartoon representing a cross section through the thoracic aorta as indicated in **(A)** with TAK on the left, normal in the middle, and LV-GCA on the right.

Large vessel imaging patterns can also help differentiate between TAK and LV-GCA in patients who are at the border of age around 50 (Figure 1A). Indeed, a large imaging cohort study recently identified six patterns of LV involvement that were different between diseases (70). Favoring TAK were involvement of the abdominal aorta, renal, and mesenteric arteries; bilateral carotid and subclavian arteries; and isolated left subclavian artery. Favoring GCA were involvement of bilateral axillary and subclavian arteries; diffuse disease including the aorta and its proximal branches; and minimal disease without clear pattern. Additionally, vascular damage with stenosis, aneurysm, or occlusion is more common in TAK while vascular inflammation alone is more common in GCA (70). Extracranial carotid arteries involved in cranial-GCA are rarely affected by TAK. Scans obtained for other reasons may also incidentally reveal CIA in the arch and descending thoracic aorta (14).

Imaging has been instrumental to define GCA but provides little insight into pathophysiology. EULAR recommends using ultrasound and MRI to diagnose cranial-GCA, with no preference in technique for LV-GCA. The optimal use and interpretation of LV imaging in clinical practice is rapidly evolving and is thus far uncertain.

## Histopathology

Normal arteries have three layers separated by dense elastic fibers (Figure 1B). From the lumen outward, these include the tunica intima, internal elastic lamina (IEL), tunica media, external elastic lamina, and tunic adventitia. Intima and media are predominantly composed of endothelial cells and vascular smooth muscle cells (VSCM), respectively (71). Their thickness and complexity increases in large elastic vessels with proportional increase in stromal cells and extracellular matrix, especially within the elastic lamellae-rich media (71). The adventitia contains a dense network of elastin and collagen connective tissue produced by fibroblasts and is interdigitated with progenitor cells, adrenergic nerves, and immunosurveillant tissue resident myeloid cells called vascular dendritic cells (vasDC) (72, 73). It is also the site of the vasa vasorum, a microvascular network composed of endothelial cells and pericytes that supplies oxygen and other nutrients to the vascular wall (72). As large arteries require increased nutritional support due to their size, vasa vasorum extend further into the media in these vessels. Outside the artery proper lie more connective tissues supported by a network of small non-muscular blood vessels (74).

### Temporal Arteries

GCA is a multi-focal, segmental destructive panarteritis (75–78) (Figure 1B). There is a transmural inflammatory infiltrate with greatest density between the adventitia and media that is composed predominantly of CD4^+^ T cells and macrophages, with few B cells and eosinophils; mature neutrophils are rare and when abundant suggest an alternative diagnosis (79, 80). Despite the name, giant cells themselves occur to a variable degree and are prominent in ~50% cases at the intima-medial junction around a deranged IEL. In the media there is laminar necrosis with loss of VSCMs and neoangiogensis; fibrinoid necrosis does not occur (79). The intima has features of vascular remodeling with hyperplasia and fibrosis, and occasionally thrombosis and recanalization, especially above sites of active inflammation. More active disease has a more diffuse and intense inflammatory infiltrate and greater number of giant cells, while quiescent disease has a scant infiltrate with fewer giant cells (81). At the end of this spectrum is “healed arteritis,” when the features of vascular damage and remodeling are seen in the absence of inflammatory cells (78).

Three other patterns of inflammation associated with GCA and PMR have also been described, together referred to as “restricted inflammation” (RI). These include small vessel vasculitis (SVV) involving the vessels in connective tissue beyond the adventitia, vasa vasorum vasculitis (VVV), and inflammation limited to adventitia (ILA) (82). Giant cells and granulomas are not present. In their limited description, SVV consisted of slightly more T than B cells and few macrophages (74), while VVV showed approximately equivalent infiltration of T cells, B cells, and macrophages (83). The extent to which these patterns are reported and their role in GCA diagnosis is controversial (82, 84). Recently, a retrospective clinicopathologic study with advanced imaging found that patients with RI had fewer cranial symptoms, less systemic inflammation, and less halo sign on ultrasound (82). However, there was no difference in visual symptoms including permanent vision loss or the degree of LV involvement between RI and classic GCA. In an accompanying systematic literature review, the positive predictive value for RI was 23%, highest for ILA with 67% for GCA and 95% for PMR (82). Notably, other forms of vasculitis, infection, and certain hematologic malignancies can present with RI and particularly with SVV (85, 86).

### Large Vessels

Large vessel pathology in GCA is less well-studied. Historically, patients with LVV have often been described to have TAK and patients with CIA may be aberrantly reported as having GCA (87). However, pathologic characteristics of GCA can be disentangled by the few studies that concurrently report TAB, which confirm histopathology is largely the same across vessel sizes (4, 81, 88, 89). Compared to TAB, the aorta has reduced adventitial inflammation with the majority of inflammatory infiltrate in the media; mild adventitial fibrosis is also occasionally seen (4, 88) (Figure 1C). Interestingly, patients with aortic dissection tend to have more diffuse involvement than seen on necropsy, suggesting more robust aortic inflammation in these patients (4, 89). CIA is histopathologically identical to GCA (14, 88). Few studies have directly compared TAK and GCA histopathology, but TAK has more inflammation and fibrosis in the adventitia and media, resulting in thicker walls (40, 48, 88, 90). There is also increased invasion of CD8^+^ T cells, B cells, and γδT cells and more giant cells compared with GCA (48, 91) (Figure 1C).

## Pathophysiology of GCA

Clinical features, epidemiology, imaging, and conventional histology give important information about GCA, PMR, TAK, and CIA, but little insight into pathophysiology. For that, we must rely on a small number of techniques, each with its own strengths and limitations, predominantly based on the characterization and manipulation of patient-derived peripheral blood mononuclear cells (PBMCs) and TAB tissue. Based on our review of data from these studies, here we envision the sequence of events that occurs in GCA, starting with initial immune activation, followed by arterial infiltration, damage, and repair response. We propose the following general model of the pathophysiology of GCA: Overlapping patterns of activation in circulating PBMCs seen between GCA and PMR suggest that immune activation precedes vascular damage. Pathologic analysis suggests vascular damage initiates in the adventitial vasa vasorum microvasculature because inflammation is never restricted to intima (85). The initial trigger for vascular injury in GCA is unknown but appears to involve interactions between pathologically activated circulating cells, especially CD4^+^ helper T cells and monocytes, and multiple vascular cell types. Upon breach of vascular immunoprivilege, recruitment of these abnormal monocytes and CD4^+^ T cells, especially IFN-γ-producing Th1 cells, cooperate to mediate vascular injury and repair. The sequence of recruitment is also unknown, but once initiated, multiple interconnected positive feedback loops sustain it in the vasculature and also likely feedback to amplify systemic immune activation.

### Immune Activation and Circulating Leukocytes

Systemic inflammation is a core feature of GCA and PMR that is largely driven by IL-6, with elevated plasma levels in both conditions (92, 93). IL-6 is a pleotropic cytokine professionally produced by monocytes, macrophages, and dendritic cells within the immune system—as well as by other cells including endothelial cells, VSMCs, fibroblasts, and B cells—as an early signal of tissue damage (94, 95). Monocytes appear to be the primary source of IL-6 among PBMCs of patients with GCA and PMR (93, 96), though the contribution from other non-circulating cell types has not been assessed and is likely significant. In the immune system, IL-6 has a key role in CD4^+^ helper T cell differentiation, promoting the development of Th17 and T follicular helper (T_FH_) cells, while inhibiting that of regulatory T cells (T_reg_) due to opposite effects of the IL-6-induced pioneering transcription factor STAT3 in the generation of these cell types during inflammation (94, 95, 97). In the liver, IL-6 stimulates production of acute phase response proteins including CRP and fibrinogen, with resultant elevation of ESR. IL-6 levels are tied closely to clinical symptoms of GCA and are higher in patients who experience more relapses, with levels rising concurrently with symptoms during relapse (93, 98). Consistent with the negative association between systemic inflammation and cranial symptoms, patients with higher serum IL-6 have fewer ischemic complications even during relapse (98–100). Whether this reflects a biologic difference or increased clinical detection remains unclear. Beyond IL-6, other cytokines are not reproducibly systemically elevated across studies; multiple studies have shown circulating TNF and IFN-γ levels are unchanged (15, 92, 93, 101).

Patients with GCA and PMR have abnormally activated PBMCs, particularly among CD4^+^ T cells, which are skewed toward effector cells. Although unchanged in number, polarization of CD4^+^ T cells is aberrant. Both conditions share increased frequency of IFN-γ ^+^ Th1 cells and a STAT3-activation pattern with increased IL-17^+^ Th17 cells and reduced Treg (92, 101–103); IL-21^+^ T_FH_ cells are also elevated in GCA but untested in PMR. Th17 cells are stimulated by the cytokines IL-23 and IL-1β and are pathologically associated with multiple autoimmune diseases. Th1 cells develop downstream of STAT4-activating IL-12, which also stimulates their production of the signature cytokine IFN-γ, a well-known driver of granulomatous inflammation in infections such as *M. tuberculosis* (104, 105). In humans, the majority of T_FH_ cells also respond to IL-12 and can co-produce IFN-γ, like Th1 cells; these cells accumulate in a multitude of inflammatory diseases and provide B cell help. T cell production of IL-21 can also enhance cytotoxicity of CD8^+^ T cells and NK cells (106). In GCA, *in vitro* culture of patient T cells with IL-21 further engenders more Th1 and Th17 differentiation (102). Beyond polarization, GCA and PMR share an increased frequency of senescent T cells (107). Studies in PMR are more limited, but GCA patients have other evidence of increased activation. These include a shift from central memory CD4^+^ T cells to effector memory and terminally differentiated effector memory cells; higher expression of HLA-DR and NOTCH1, which has a pleomorphic pro-inflammatory function in mature T cells; and a gene expression signature enriched for T cell receptor signaling (102, 108–110).

In comparison, other circulating lymphocytes appear to be less impacted, though comprehensive assessment using high-dimensional analyses is lacking. CD8^+^ T cells from GCA and PMR patients have increased oligoclonality, and many but not all studies report lower numbers; at least in GCA, they also express higher HLA-DR (102, 111–115). In some studies, there is a global reduction in the number of B cells, while NK cell numbers are unchanged (102, 116).

In the myeloid compartment, prominent changes include increased numbers of circulating monocytes and immature neutrophils. Monocytosis of classical CD14^bright^ CD16^lo^ cells is present in both GCA and PMR (93, 117). In GCA, these cells are phenotypically identical to healthy controls (118, 119). However, they are transcriptionally primed in circulation, expressing higher levels of pro-inflammatory cytokines *IL6, IL1B*, and IL-12/23 components *IL23A* (p19)*, IL12A* (p35), and *IL12B* (p40) as well as extracellular matrix-degrading (ECM) gelatinases *MMP2* and *MMP9* (96, 101, 118). Surprisingly, left shift with increased circulating immature neutrophils was recently shown to be the major cellular difference by mass cytometry (CyToF) between untreated GCA patient and healthy control PBMCs (80). Collectively, these data suggest there is increased bone marrow myelopoiesis and/or recruitment in active disease. Whether PMR patients have the same transcriptional changes to monocytes or cellular distribution remains to be seen.

### Initiating Arterial Inflammation

Two major challenges to understanding GCA pathogenesis are the absence of a commonly used mouse model and the lack of availability of sequential patient samples. Given these challenges, mechanistic insights rely on three human systems based on temporal artery: (1) observations from TAB; (2) manipulation of TAB or normal arteries in Matrigel (120); or (3) manipulation of TAB or normal arteries in chimeric mouse systems (121). The chimeric systems are the most complex and have evolved over time. One currently used system involves three sequential steps to produce inflammation, which may rely in part on alloreactivity (“subcutaneous-chimera”): (1) implantation of a human artery segment as a subcutaneous graft on the lower midback of a highly immunocompromised NOD.Cg-*Prkdc*^*scid*^ *Il2rg*^*tm*1*Wjl*^/SzJ (NSG) mouse that lacks all lymphocytes and has severely defective myeloid cells; (2) stimulation with lipopolysaccharide (LPS), activating xenograft vasDC to adopt a pro-inflammatory CD83^+^ CD86^+^ phenotype and produce T-cell recruiting chemokines; and (3) adoptive transfer of PBMCs from treatment-naïve allogeneic GCA patients, generating immune infiltration that histologically and transcriptionally resembles GCA (103, 121). Additional mechanistic insights can also be learned from another chimeric vascular allograft rejection model, where human coronary artery xenograft surgically replaces the mouse infrarenal aorta (“interposition-chimera”) (122). Similar to subcutaneous-chimeras, this model uses highly immunocompromised CB17.Cg-*Prkdc*^*scid*^*Lyst*^*bg*−*J*^/Crl mice that lack T and B cells through the same *Prkdc* mutation but differ in the mechanism of impaired NK and granulocyte function. After adoptive transfer of PBMCs from allogeneic blood donors, these mice develop xenograft vascular inflammation even in the absence of LPS over a similar time course. However, inflammation is more histopathologically similar to TAK, with prominent adventitial and intimal CD4^+^ and CD8^+^ T cell invasion and hyperplasia; unlike TAK and GCA, very few leukocytes invade the media, myeloid infiltration is rare, IEL are preserved, and neovascularization does not occur (123).

Arterial invasion requires activation of both circulating and vascular cells. Emphasizing the importance of pathogenic circulating cells, in subcutaneous-chimeras, normal human PBMCs cannot typically invade artery grafts even in the presence of LPS (109). Meanwhile, GCA-derived alloreactive T cells cannot invade artery grafts in the absence of LPS (121). Some of these effects may be caused by recruitment and duration of contact with the subcutaneous graft because normal human T cells—but not myeloid cells—can invade the interposition-chimera allograft without further stimulus downstream of endothelial antigen presentation to CD4^+^ and CD8^+^ T cells (122, 124). Interestingly, upon co-implantation of TAB fragments from GCA, PMR, or control patients into NSG mice, T cells recirculate from GCA arteries and invade PMR but not normal arteries in the absence of LPS, suggesting PMR vessels have lost immunoprivilege (121). PMR vasDC have a partially activated CD83^+^CD86^−^ phenotype on TAB. However, whether vasDC cause the arterial leakiness in PMR or GCA vessels is unclear because adoptive transfer of GCA T cell alloreactive clones in subcutaneous-chimeras in the absence of LPS also induces a CD83^+^ vasDC phenotype, but T cells do not invade (121). Thus, while LPS stimulation can breach immunoprivilege in transplanted arterial sections *via* vasDC activation and perhaps prolonged contact with allogeneic T cells, this may not be the initiating event of vascular damage in GCA. These data also suggest—despite some overlapping phenotypes in PBMCs—that pathogenic differences occur between GCA and PMR cells that can facilitate entry into PMR primed vessels.

IFN-γ can independently break vascular immunoprivilege. Incubation of normal artery in Matrigel with IFN-γ induces VSMC expression of several chemokines including monocyte-recruiting CCL2 as well as Th1- and CD8^+^ T cell-recruiting CXCL9, CXCL10, and CXCL11 (125). It also induces VSMC ICAM-1 expression, an adhesion molecule that binds leukocyte integrins and, when expressed by endothelial cells, facilitates vascular transmigration. Remarkably, addition of healthy control PBMCs results in invasion of macrophages—but not healthy T cells—that subsequently become giant cells (125). Endothelial expression of HLA-DR is also induced by IFN-γ (122, 124), but whether pathogenic GCA PBMCs facilitate T cell entry has not been tested. This axis may also be important in early vascular injury in GCA, as TAB shows increased ICAM-1 expression by VSMCs in regions of structurally normal skip lesions and as well as by endothelial cells in the vasa vasorum (126, 127). Notably, other cytokines such as macrophage-derived TNF and IL-1β can also induce ICAM-1 expression on endothelial cells *in vitro* as well as enhance its upregulation by IFN-γ (128). Interestingly, *IL1B* is increased in PMR TAB compared to controls (129). Thus, local induction of cytokines from activated circulating cells may similarly prime segments of vasculature for inflammatory cell entry, though the mechanism of tissue tropism to the LV vasculature with this lens remains to be explored.

### The Feed-Forward Inflammatory Infiltrate

On TAB, activated memory CD4^+^ T cells massively invade GCA arteries, where they polarize even further into effector cells compared to PBMC, homing mostly to the adventitial-medial border but present in all three layers. These express a broad repertoire of T cell receptors with a minimal degree of clonal expansion (130, 131). While a comprehensive assessment of infiltrating T cells is lacking, there are varying degrees of IFN-γ, IL-17, IL-21, and IL-9 produced. The balance of polarization differs between patients and is functionally relevant because cranial ischemic symptoms correspond to increased Th1 function on TAB (99, 132, 133). Indeed, ischemia positively correlations with: (1) the Th1 signature cytokine IFN-γ, (2) its activator IL-12p35, and (3) the downstream number of giant cells (99, 132, 133). In contrast, patients with higher expression of the Th17 signature cytokine IL-17A have fewer relapses and more systemic symptoms (134, 135). Consistent with this, in interposition-chimeras, IL-17 blockade does not impact intimal hyperplasia but does reduce *IL6* (136). Though minimally described, TAB with RI also reflect different T cell composition. Compared to transmural inflammation, SVV has low levels of IL-17 and intermediate levels of IL-9 while VVV/ILA has the opposite pattern (135); the distribution of IFN-γ has not been described. VVV further lacks NOTCH1^+^ infiltrating T cells (109). Thus, T cell polarization differs between clinical and pathologic phenotypes, but how different signature cytokines affect pathogenesis largely remains to be explored.

Myeloid cells also diffusely infiltrate all three layers of the artery on TAB and densely populate granulomas around the IEL. These include three populations: a smaller CD16^−^CCR2^+^ CX3CR1^−^ cells that produce IL-6 and IL-1ß and phenotypically resemble circulating monocytes; CD16^+^CCR2^−^CX3CR1^+^ macrophages that produce MMP9, MMP2, VEGF, and the potent mesenchymal mitogen PDGF; and giant cells that functionally overlap with CX3CR1^+^ macrophages but express the above effectors to an even greater degree by immunohistochemistry (96, 117, 137, 138). Other myeloid generated cytokines elevated in TAB that contribute to the pro-inflammatory environment include TNF, IL-12, and IL-23 (101, 139, 140). Co-culture of human peripheral blood monocytes with aortic adventitial fibroblasts induces their differentiation into macrophages that produce MMP9 (141). In other biologic conditions, various cytokines can stimulate macrophage fusion into giant cells—including IFNγ, IL-1β, and IL-6—but correlation between IFN-γ levels and number of giant cells on TAB suggest this is the primary mechanism in GCA (99, 142). Collectively, these data suggest monocytes that are transcriptionally primed to produce pro-inflammatory cytokines and gelatinases in circulation are recruited from the peripheral blood, differentiate in the inflamed vessel into macrophages, and further combine to form giant cells in response to IFN-γ. However, it is also possible that monocytes and macrophages are independently recruited to inflamed vessels from the circulation.

Multiple cell types generate positive feedback chemokine loops that enhance T cell and myeloid recruitment. In subcutaneous-chimeras, vasDC produce CCL19 and CCL21 as well as its receptor CCR7, trapping them in the artery upon activation (73). They also produce CCL20 and attract cells expressing CCR6, a phenotype shared by many infiltrating T cells on TAB as well as by Th17 and Th1/Th17 precursors in GCA patient peripheral blood (92, 143, 144). Notably, a variety of cells including DC, macrophages, Th17 cells, and VSMC can produce CCL20 and while it is overexpressed on TAB, the cellular source has not been shown (134, 136, 145). VSMC are a nexus for accentuating inflammatory signals: macrophage-generated TNF stimulates macrophage-attracting CX3CL1 *in vitro*; macrophage-expressed PDGF induces monocyte-recruiting CCL2 in Matrigel; Th17-produced IL-17 causes Th1/Th17-recruiting CCL20 *in vitro* and in interposition-chimeras; and Th1-derived IFN-γ provokes CX3CL1 plus Th1-, CD8^+^ T cell- and monocyte-recruiting chemokines, as previously described (125, 136, 146, 147). Finally, when co-cultured, fibroblasts induce monocyte expression of monocyte-recruiting CCL2 (141). Thus, upon entry of T cells and monocytes in the blood vessel, interactions with resident vascular cells perpetuate inflammation.

T cell interactions with other vascular cells also enhance inflammation. Endothelial cells in the vasa vasorum pathologically express Jagged1 on TAB. This can be experimentally reproduced *in vitro* by GCA plasma and mitigated by anti-VEGF, consistent with the increased systemic levels of VEGF in patients with GCA. *In vitro* and in subcutaneous-chimeras, Jagged1 ligates NOTCH1 expressed by circulating T cells and enhances their polarization to Th1 cells and, to a lesser extent, Th17 cells (103, 109). Upon entry into the vessel, T cells interact with vasDC. In normal artery specimens, these constitutively express PD-L1, a molecule that restrains PD-1^+^ T cells generated during chronic immune stimulation (148). In GCA TAB, vasDC upregulate antigen presentation machinery of HLA-DR, CD83, and CD86 but downregulate PD-L1; meanwhile, vascular invasive but not circulating T cells highly express PD-1 (73, 121, 148). Blocking PD-L1 in subcutaneous-chimeras results in exuberant inflammation, suggesting physiologic PD-1L^+^ vasDC restraint is lost in GCA immunopathology (148). Thus, inflammatory changes to other cell types augment the pathogenicity of pre-activated T cells.

### Vascular Injury and Repair

Macrophages drive vascular injury largely *via* gelatinases. In normal vessels, VSMC constitutively produce pro-MMP2 and its inhibitor TIMP2 resulting in a quiescent vessel without proteolysis (149). With inflammation, macrophage- and giant cell-derived MMP2 and MMP9 outpace inhibitors, resulting in progressive degradation of ECM that is consequently more easily infiltrated by T cells (118, 149). Destruction occurs locally around macrophages as demonstrated by the restriction of MMP9 and proteolysis to the adventitia in ILA on TAB (118). Giant cells are gelatinase factories and, taking residence along the IEL, cleave and destroy it. The mechanisms that drive VSMC laminar necrosis are poorly described, but likely also involve myeloid mediators because, like IEL degradation, it does not occur in interposition-chimeras that lack myeloid recruitment (122, 123).

Macrophages and Th1 inflammation launch vascular remodeling, resulting in intimal hypertrophy and neovascularization. In response to a variety of mitogenic signals in Matrigel but most robustly to PDGF, healthy contractile VSMC become proliferative, invasive myointimal cells (146). These leave the media and invade the intima where they produce the vascular ECM proteins collagen I and III, generating the hypertrophic neointima (138, 146). TAB levels of PDGF and IFN-γ correspond to the degree of intimal hyperplasia, which in turn correlates with ischemic symptoms as the macrolumen becomes progressively stenotic (138). While macrophages and giant cells at the media/intimal border both produce PDGF, recombinant IFN-γ can also directly stimulate VSMC to produce PDGF as well as upregulate its receptor in interposition-chimeras that lack PBMC adoptive transfer, resulting in neointimal hyperplasia even in the absence of cellular infiltration (150). Both macrophage and T cell pathways are likely active in GCA. Patients with ischemic symptoms also have higher plasma levels of endothelin 1 (ET-1), a potent vasoconstrictor physiologically generated by endothelial cells. Interestingly, ET-1 is expressed by infiltrating immune cells on TAB and can redundantly generate intimal-invasive myointimal cells from VSMC in Matrigel (151). The degree of intimal hyperplasia further correlates with the degree of neovascularization in the intima and media, and in turn, to levels of VEGF on TAB, suggesting this process is driven by hypoxia (152). However, neovascularization co-localizes with macrophage- and giant cell-rich areas on TAB (152). Thus, the extent of neovascularization likely reflects the degree of macrophage and giant cell activation through multiple mechanisms including their production of VEGF. Collectively, vascular remodeling results in thickened blood vessels that cause symptomatic ischemia and generates a conduit for further inflammatory cell entry through leaky neovasculature (127).

### Treatment Effects

Glucocorticoids are the standard therapy for GCA and PMR. Consistent with the need for higher doses in GCA than PMR, systemic changes occur first while local changes seen in TAB generally take much longer. Plasma IL-6 is strongly inhibited after a single dose of GC, but the median time to normalization is 4 weeks (93). Though systematic sequential immunophenotyping of PBMCs during treatment has not been reported, B cells appear to be the first to respond and normalize after 2 weeks, a time course consistent with changes in mobilization (116). After 3 months of treatment, Th17 cell frequency, and CD4^+^ but not CD8^+^ T cell HLA-DR expression return to normal (92, 101, 102). Monocyte numbers are also reduced at this time but remain higher than healthy controls (117). Furthermore, after 3–9 months of treatment, monocyte expression of *IL6* and Th17-activating *IL1B* and *IL23A* normalize while expression of Th1-inducing and activating *IL12A* and *IL12B* remain elevated (101). Consistent with this, among CD4^+^ T cells, Th1 take longer to respond, normalizing with full disease remission (101, 102). Finally, CD8^+^ T cell numbers take up to 2 years to return to baseline numbers (111). Indeed, in patients diagnosed with GCA for at least 2 years, increased circulating CD4^+^ T cells, reduced CD8^+^ T cells, and the corresponding increased CD4^+^/CD8^+^ ratio but not inflammatory markers or monocytes numbers have recently been shown to be associated with thoracic aortic dilatation compared to controls (22).

Reports of TAB re-biopsy after GC treatment reveal similar results to PBMCs with initial control of Th17 pathways, and later reduction in Th1 pathways, as well as a prolonged timecourse of vascular healing. Compared to TAB with active GCA, re-biopsies at 3-9 months phenocopy peripheral blood and show profound reduction in *IL6, IL1B, IL23A*, and *IL17* while *IL12A IL12B*, and *INFG* are unchanged (101). In another study, patients with paired re-biopsy at 1 year demonstrated a global reduction in all tested cytokines including *IL1B, IL6, IL23A, IL12A, IL12B*, and *IFNG* as well as *MMP9*, though patients with more relapses showed higher levels of *IL12B* and *IFNG* (139). Consistent with this prolonged time course, a prospective study of 40 patients re-biopsied at 3, 6, 9, and 12 months found active arteritis in 7/10, 9/12, 4/9, and 4/9 samples, respectively, despite normalization of inflammatory markers and clinical symptoms. There was also a time-dependent increase in vascular remodeling (153). Thus, GC quickly control Th17 signatures in circulation and TAB, likely reflecting loss of STAT3 activation from monocyte-derived IL-6. Meanwhile, Th1 pathway takes longer to respond, consistent with prolonged monocyte production of STAT4-activating IL-12, which may drive relapse and ongoing vasculitis in some individuals. Finally, vascular remodeling continues after active inflammation resolves, like the prolonged FDG signal on PET imaging.

Multiple other treatment modalities have been tested for GCA in randomized clinical trials. In the GiACTA trial, targeted blockade of IL-6R with TCZ demonstrated superiority to a course of GC alone in achieving steroid-free remission at 1 year, as defined by lack of clinical flare and normal level of IL-6-induced CRP, becoming the first non-steroid FDA-approved treatment for GCA (11). Interestingly, fewer patients with relapsing disease responded to TCZ than patients with untreated disease, raising the possibility that these patients may have more Th1 driven disease. Furthermore, one patient in the TCZ every-other-week arm developed the ischemic complication of anterior ischemic optic neuropathy. In another study, a patient with highly active disease that normalized on TCZ—but who died unrelated to GCA after 6 months of therapy—had widely active vasculitis on autopsy (154). Though GCA-related adverse events were not statistically different between groups in GiACTA, these raise the question if TCZ controls vascular-level inflammation or if it blocks systemic manifestations of flare, which may further differ between newly diagnosed and relapsed patients.

Further insights to this question are suggested by the differing results of the two open-label studies of anti-p40 ustekinumab. In the initial promising Irish trial, all patients recruited had relapsing disease and ustekinumab was successful in achieving GC-reduction without flare, albeit with persistent low GC dose in the majority of patients. Notably, of 10 patients with LV disease in this study, eight underwent reimaging by CT angiography, which demonstrated not only a halt to further vascular damage but improvement of wall thickening in all patients and complete resolution in four (49). In the American trial, both newly diagnosed and relapsing patients were recruited and all patients were required to end GC at 6 months, resulting in clinical flare across the majority of patients with elevated inflammatory markers and PMR symptoms; though data was not shown for relapse between newly diagnosed vs/ relapsing patients they were stated not to be different (50). Vascular imaging follow up was not reported (50). Consistent with molecular studies, these differing trial results suggest a degree of independence between systemic symptoms of flare downstream of IL-6 and vascular damage in relapsing patients downstream of IL-12. While GC controls both endotypes, targeted therapies directed at either can fail; however, only low dose GC may be required to control the IL-6 axis, as in PMR, at least in patients with relapsing disease. These data also emphasize that long-term follow up of TCZ-treated patients and further clinicopathologic correlation will be important and the utility of a randomized trial for ustekinumab in GCA patients with relapsing disease. Similar to GC, blocking STAT activation directly with JAK inhibitors would allow combinatorial blockade of IL-6 and IL-12/23 without GC side effects and is theoretically compelling. Indeed, multiple JAK inhibitors are currently in clinical trials (12).

Beyond IL-6, another potential emerging treatment is to target T cell overactivation directly. Indeed, abatacept (CTLA-4:Fc) was superior in achieving relapse-free survival at 1 year in a phase 2 trial (155). Other targeted therapies using TNF blockade with infliximab, adalimumab, or etanercept have been ineffective (156–158).

### Comparison to TAK and CIA

**Figure 2:**
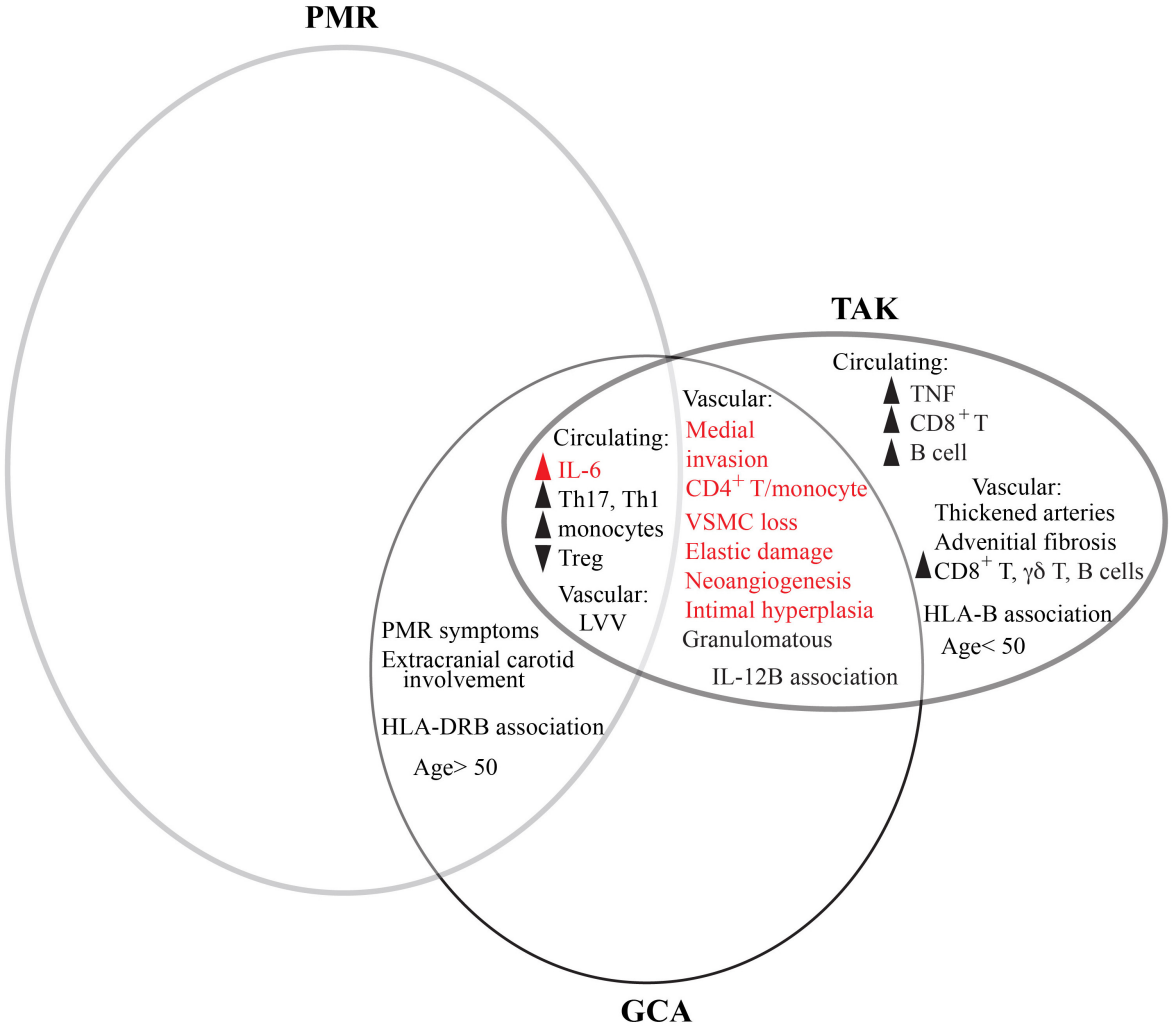
Comparative features between GCA, PMR, and TAK. PMR and GCA are overlapping clinical entities as patients can concurrently have both conditions. However, more patients with GCA have PMR than do patients with PMR have GCA. Meanwhile, GCA and TAK share features of vascular inflammation in large vessels. PMR, GCA, and TAK all share elevated levels of IL-6 and changes to circulating immune cells. However, distinguishing TAK from GCA are some circulating lymphocytes and cytokines, vascular pathology, and patient age. Many features are common to other types of vascular disease, such as AAA (red). There is not currently enough information to determine the extent to which CIA overlaps with other conditions.

Due to relative lack of tissue compared to GCA, less is known about the immunopathology of TAK, and that of the more recently-described entity CIA remains virtually unexplored. Consistent with overlapping but distinct pathology, TAK shares several features in common with GCA but differs in cytotoxic mediators (Figure 2). Like GCA, changes to circulating inflammatory cells also reflect those in the tissue. Systemically, patients with TAK share elevated systemic levels of IL-6 and increased circulating classical monocytes, Th17 cells, and Th1 cells with fewer Treg (159–161). In tissue, memory CD4^+^ T cells—including Th1 and Th17 subsets—are likewise the most prevalent invasive cell type, with equal macrophage infiltration between conditions (48, 162). Interestingly, in the opposite pattern of GCA, peripheral Th1 cells respond better to steroids than Th17 cells, which remain elevated despite clinical remission (162). Unlike GCA, patients with TAK also have elevated systemic TNF and consistent with this, TNF inhibitors are at least modestly clinically effective (161, 163). The major difference between TAK and GCA is among non-CD4^+^ lymphocytes, as B cells and CD8^+^ T cells are elevated in peripheral blood and tissue. As suggested by genetic HLA class I associations, CD8^+^ T cells seem particularly relevant, rising in circulation during flares and found actively killing vascular cells on electron microscopy (91, 159). Interestingly, GCA patients with relatively higher levels of CD8^+^ T cell invasion also have more severe disease, though in this condition it may also reflect the degree of Th1 inflammation given mutual dependence of CD8^+^ T cells on the positive-feedback IFN-γ-CXCR3 recruitment loop (114).

## Perspectives and Future Directions

GCA is a complex disease because it lies at the interface of two clinical spectra—the pathologically similar granulomatous vasculitides and the clinically overlapping GCA and PMR—each of which have historically been imprecisely defined based on clinical phenotypes and therefore often overlap in the literature (Figure 2). Additionally, emerging results from advanced imaging and pathologic analysis show two additional spectra—LV- and cranial-GCA and histologic RI—that demonstrate even greater overlap with PMR than previously recognized. Compounding this complexity is the clinical need to treat GCA emergently and the recent transition from pathology to imaging for diagnosis, which respectively limit the availability of untreated patient PBMCs and tissue specimens for research.

Despite phenotypic similarities between TAK and GCA, the multiple differences between affected patients—in age, demographics, vascular distribution, genetics, histopathology, and immunophenotype—suggest that these are distinct disease entities with some degree of convergence (Figure 2). Furthermore, most shared features between TAK and GCA are not unique to vasculitis. In fact, the common vascular condition of abdominal aortic aneurysm (AAA), a permanent dilatation to the aorta that affects 1–2% of men age 65 and 0.5% of women age 70, shares most features: elevated systemic levels of IL-6; monocytosis; medial invasion of memory CD4^+^ T cell and macrophages; and vascular remodeling with dissolution of the elastic lamellae, loss of VSCM, and neoangiogenesis (164–169) (Figure 2). This suggests despite different triggers of vascular injury, many pathways of arterial damage converge, though some differences persist and may inform our understanding of disease mechanisms. For example, granulomatous inflammation likely reflects the higher vascular IFN-γ and macrophage invasion in GCA, CIA, and TAK compared to AAA, while changes to circulating T cells reflect higher systemic levels of IL-6 (164, 167, 169, 170). Likewise, the prominent fibrosis in TAK appears to be an important distinction and may represent a novel disease target. Interestingly, the comparison of AAA is particularly relevant for GCA as they share several other epidemiologic features, including old age with rare incidence below age 50, increased prevalence in Northern Europe, and smoking as a core risk factor for aneurysm development (24, 166). Thus, these unexplained risk factors in GCA may represent common mechanisms of vascular risk.

The heterogeneous and overlapping patterns of pathologic RI, LV-involvement, and PMR with GCA remain a mystery. One possibility is that RI and PMR represent more subtle degrees of vascular injury that jointly affect the microvasculature, including that of large arteries. In some patients who experience an unknown stimulus, this may progress to more fulminant disease. Supporting this, a recent report demonstrated a key role for NOTCH3 signaling between the arterial microvasculature and synovial sublining fibroblasts to generate synovitis in rheumatoid arthritis (171). Indeed, microvascular endothelial cells upregulate the NOTCH3 ligand Jagged1 in GCA, though whether this also occurs in PMR synovitis has not been tested (103). Furthermore, subtle microvascular changes may explain the ability of GCA T cells to recirculate into PMR arteries in early experiments (121). Alternatively, the regulatory logic of CD4^+^ T cells may differ in LV-GCA and/or RI. For example, several lines of evidence suggest that a Th1 signature favors vascular damage and ischemia. Mechanistically, this is an especially feed-forward module in the vasculature through cyclical recruitment of Th1, myeloid cells, and CD8^+^ T cells that ultimately propagates stenotic tissue remodeling through macrophage activation and giant cell formation. However, the role of other helper T cell modules such as Th17 in GCA is less clear—despite the evidence that they are also present systemically and in vascular tissue. Though it is possible circulating Th17 and T_FH_ cells may simply represent off-target STAT3 activation of IL-6, another possibility is that this module corresponds more to LV inflammation. Supporting this, patients with LV disease typically have more systemic symptoms, as do patients with increased IL-17 on TAB. Furthermore, mice lacking the Rac activator Def6, a negative regulator of IRF4, spontaneously develop granulomatous aortitis due to aberrant T cell production of IL-21 and IL-17 (172). Given the more recent emphasis on LV-GCA, radio-pathologic correlation has not yet been performed but would be interesting. Comprehensive assessment of the immune infiltrate in RI is more easily achieved.

Since the discovery of GCA, the trigger for vascular inflammation has been questioned. Here, we propose a model where systemic changes in the circulation precede vascular injury and are required for disease initiation. In this model, systemic activation likely initiates in myeloid cells—perhaps monocytes—leads to circulating CD4^+^ T cell polarization downstream of the pioneering transcription factors STAT3 and STAT4. However, recently published data suggests myeloid activation may be even further upstream, as early as the bone marrow, given the prominent left shift seen by CyToF in patients with untreated GCA (80). Supporting this, GCA can occur as a paraneoplastic phenomenon to myelodysplastic/ myeloproliferative neoplasms as well as in the recently described somatic, myeloid-activating autoinflammatory condition VEXAS (173, 174). Upon breach of vascular immunoprivilege, pre-activated monocytes and CD4^+^ T cells mutually enter the vessel and cooperate to destroy it. In chimeric systems, T cell invasion likely relies on allorecognition, but in GCA, HLA associations suggest an antigenic driver that is thus far elusive and may describe tissue tropism to the microvasculature of large vessels. With GC treatment, the IL-6-STAT3 axis regulating systemic symptoms is more quickly controlled while IL-12-STAT4 axis mediating vascular damage, at least in TAB, requires prolonged treatment, consistent with the different time course needed to control these cytokines in circulating monocytes. In the absence of IL-6, Th1 cells may paradoxically initially increase as the cytokine microenvironment favors their generation and persistence (105), consistent with the weaker performance of TCZ in relapsed patients and potentially better performance by ustekinumab (11, 49). Whether flares represent reemergence of abnormally activated circulating myeloid cells, lack of control in the vessel, or some other unexpected mechanism is unclear, but comprehensive longitudinal phenotyping is likely to be informative to this end, as recently described in rheumatoid arthritis (175). In the future, integration of such longitudinal data with imaging will be particularly useful to define clinically relevant entities such as persistent subacute inflammation, flare, and remission. Ultimately, clinical trials of various immune modulators in patients with GCA will provide further insights into proposed disease mechanisms and should include dual assessment of clinical flare as well as vascular damage.

## Author Contributions

MR, DR, and PM wrote the manuscript. All authors contributed to the article and approved the submitted version.

## Conflict of Interest

The authors declare that the research was conducted in the absence of any commercial or financial relationships that could be construed as a potential conflict of interest.
